# Phylogenetic relationship between the endosymbiont “*Candidatus* Riesia pediculicola” and its human louse host

**DOI:** 10.1186/s13071-022-05203-z

**Published:** 2022-03-05

**Authors:** Alissa Hammoud, Meriem Louni, Dorothée Missé, Sébastien Cortaredona, Florence Fenollar, Oleg Mediannikov

**Affiliations:** 1 Institut Hospitalo-Universitaire (IHU) Méditerranée Infection, 13005 Marseille, France; 2 Institut de Recherche pour le Développement (IRD), Assistance Publique-Hôpitaux de Marseille (APHM), Microbes Evolution Phylogeny and Infections (MEPHI), Aix-Marseille University, 13005 Marseille, France; 3 Department of Biology, Faculty of Sciences, M’Hamed Bougara University, 35000 Boumerdès, Algeria; 4 IRD, CNRS, MIVEGEC, Université Montpellier, 34394 Montpellier, France; 5 Institut de Recherche pour le Développement (IRD), Assistance Publique-Hôpitaux de Marseille (APHM), Vectors Infections Tropicales and Mediterranean (VITROME), Aix-Marseille University, 13005 Marseille, France

**Keywords:** Human lice, *Candidatus* Riesia pediculicola, Co-evolution, Mitochondrial genes, Housekeeping genes, Phylogenetic analysis

## Abstract

**Background:**

The human louse (*Pediculus humanus*) is a haematophagous ectoparasite that is intimately related to its host. It has been of great public health concern throughout human history. This louse has been classified into six divergent mitochondrial clades (A, D, B, F, C and E). As with all haematophagous lice, *P. humanus* directly depends on the presence of a bacterial symbiont, known as “*Candidatus* Riesia pediculicola”, to complement their unbalanced diet. In this study, we evaluated the codivergence of human lice around the world and their endosymbiotic bacteria. Using molecular approaches, we targeted lice mitochondrial genes from the six diverged clades and *Candidatus* Riesia pediculicola housekeeping genes*.*

**Methods:**

The mitochondrial cytochrome* b* gene (*cytb*) of lice was selected for molecular analysis, with the aim to identify louse clade. In parallel, we developed four PCR primer pairs targeting three housekeeping genes of *Candidatus* Riesia pediculicola: *ftsZ*, *groEL* and two regions of the *rpoB* gene (*rpoB*-1 and *rpoB*-2).

**Results:**

The endosymbiont phylogeny perfectly mirrored the host insect phylogeny using the *ftsZ* and *rpoB*-2 genes, in addition to showing a significant co-phylogenetic congruence, suggesting a strict vertical transmission and a host–symbiont co-speciation following the evolutionary course of the human louse.

**Conclusion:**

Our results unequivocally indicate that louse endosymbionts have experienced a similar co-evolutionary history and that the human louse clade can be determined by their endosymbiotic bacteria.

**Graphical Abstract:**

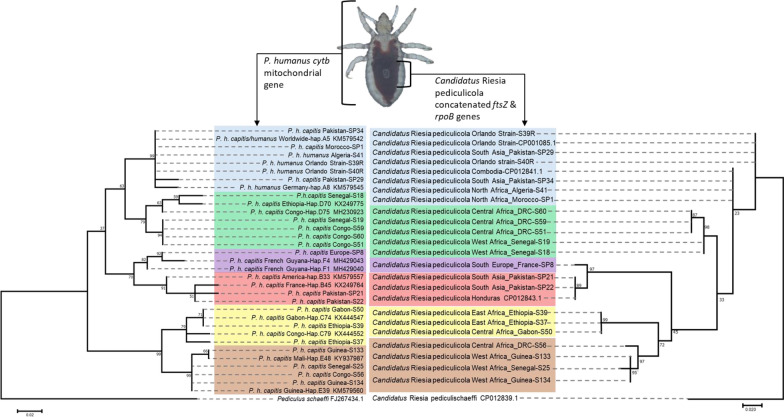

**Supplementary Information:**

The online version contains supplementary material available at 10.1186/s13071-022-05203-z.

## Background

The human louse, *Pediculus humanus* (Phthiraptera: Anoplura), has been a great public health concern throughout human history. It is one of the most ancient haematophagous ectoparasites and intimately related to its host [1]. Two ecotypes can infest *Homo sapiens*: *Pediculus humanus corporis* and *Pediculus humanus capitis. Pediculus h. corporis*, known as the body louse, infests people living in poor hygienic conditions and is the principal vector of *Rickettsia prowazekii* (epidemic typhus agent), *Borrelia recurrentis* (relapsing fever agent), *Bartonella quintana* (trench fever agent) [2, 3] and probably *Yersinia pestis* (pandemic plague agent) [4]. *Pediculus h. capitis*, known as the head louse, has a widespread infestation rate regardless of the hygiene conditions [5, 6]. However, its capacity to be a potential vector of disease remains poorly understood [7]. The genetic diversity of human lice has been extensively investigated using mitochondrial (mt) genes [cytochrome* b* (*cytb*), cytochrome oxidase subunit 1 (*cox1*) and 12S ribosomal RNA (*12S*)], allowing their classification into six divergent clades that may be grouped in three sister groups (A–D, B–F and C–E), each exhibiting a specific geographical distribution [8]. Clade A is the most prevalent, with a worldwide distribution, while clade D is only found in central Africa. Clade B is reported on all continents, while the genetically close clade F has a geographically restricted distribution and has been recently reported in South America. In addition, clade C has been identified in lice from central Africa and Asia, whereas clade E is reported in central and west Africa [6–9].

Like all haematophagous lice, *P. humanus* directly depends on the presence of endosymbiotic bacteria to supplement its unbalanced diet and metabolic integration [9]. Symbiosis is a common and widespread phenomenon that has a major effect on the biology of haematophagous arthropods. This interaction encompasses a whole range of possible symbiotic associations, ranging from strict parasitism to obligate mutualism [10]. Body and head lice host the same primary endosymbiotic bacterium, *Candidatus* Riesia pediculicola [9], which is classified in the genus *Candidatus* Riesia (class *Gamma-Proteobacteria*, family *Enterobacteriaceae*) [11, 12]. The symbionts are transovarialy transmitted to progeny and are housed in the mycetome, which is localized on the ventral side of the louse midgut. Migration is initiated by a stimulating factor associated with the adult moult. In females, the symbionts migrate to the lateral oviducts at the beginning of oogenesis, and in male adults, the stomach disc will degenerate over time [13–15]. A genomic study of the human body louse and its primary endosymbiont has provided new insights into *Candidatus* Riesia pediculicola [11]. This bacterium has a small genome (< 600 genes) containing a panel of genes encoding for the synthesis of essential B-group vitamins that are crucial to the host’s diet [16, 17]. Indeed, the symbiont supplements the host’s diet with thiamine, riboflavin, niacin, pantothenic acid, pyridoxine, biotin and folate (B1, B2, B3, B5, B6, B7 and B9 vitamins, respectively) [16–18]. Removal of the mycetome from *Pediculus* females leads to their death a few days later, as well as to the production of deformed eggs [10, 16]. It is a distinct possibility that the development of transgenic lineages of host and symbiont genes will facilitate our understanding of host–symbiont function and integration [19].

Human and chimpanzee lice (*Pediculus schaeffi*) diverged from a common ancestor, as did their human and chimpanzee hosts (*Pan troglodytes*), respectively, sometime between approximately five and seven million years ago. Interestingly, *Candidatus* Riesia pediculicola shared a common ancestor with the *P. schaeffi* endosymbiont (*Candidatus* Riesia pediculischaeffi) roughly 5.4 million years ago [17, 18]. The evaluation of this co-evolutionary association between lice and their endosymbionts might provide new insights into human evolution [17]. Also, phylogenetic studies have shown a higher sequence similarity between clade A head and body lice endosymbionts than between clade A and clade B head lice endosymbionts. These results suggest that the endosymbionts co-evolved with their hosts’ clades [17].

The aim of the present study was to establish a co-evolutionary relationship between the endosymbiotic bacteria and their human lice hosts from different clades using molecular approaches. We investigated mt genes from the six divergent clades of human lice and the housekeeping genes of *Candidatus* Riesia pediculicola in order to determine the louse clade using its endosymbiont bacteria population.

## Methods

### Lice selection and DNA extraction

From among the human lice collection of the IHU Mediterannée Infection laboratory, we selected 126 head and body lice that had been collected from around the world to perform the molecular study (Additional file 1: Table S1). These specimens had been collected in dry tubes, transported to our laboratory and frozen at − 20 °C for subsequent analysis. Prior to DNA extraction, each louse was externally decontaminated as previously reported [20]. Each specimen was cut longitudinally, and one half was frozen for subsequent analysis. DNA was extracted using a DNA extraction kit (QIAamp Tissue Kit; Qiagen, Hilden, Germany), using the EZ1 instrument in accordance with the manufacturer’s protocol.

### Lice genotypic status

#### Haplogroup identification using qPCR assays

To identify the lice mt clades, DNA samples were subjected to clade-specific quantitative duplex real-time PCR (qPCR) targeting a portion of the *cytb* gene [21]. Each duplex is specific to clades A–D and B–C, noting that the B–C duplex also amplifies clade E lice, classified as a sub-sister clade within clade C lice. DNA amplification was performed as described previously [22]. Lice with a known clade were used as a positive control, while the master mixtures served as negative controls.

#### Haplotype identification using standard PCR and sequencing

Based on the qPCR results, we randomly selected 46 lice specimens encompassing the full range of clade diversity for phylogenetic analysis. DNA samples were subjected to standard PCR, targeting a 347-bp fragment of the *cytb* gene [23]. The final reaction volume (25 μl) consisted of 12.5 μl Amplitaq gold master mixes, 0.5 μM of each primer, 5 μl DNA template and water. *cytb* amplification was performed in the Applied Biosystems 2720 Thermal Cycler (Applied Biosystems, Thermo Fisher Scientific, Waltham, MA, USA) with the following thermal cycling profile: 1 cycle at 95 °C for 15 min; then 40 cycles of 1 min at 95 °C, 30 s at 56 °C and 1 min at 72 °C; followed by a final extension step for 5 min at 72 °C. Successful amplification was validated by electrophoresis in an 1.5% agarose gel. Amplicons were then purified on NucleoFast 96 PCR plates (Macherey–Nagel EURL, Hoerdt, France) according to the manufacturer’s instructions and sequenced using the Big Dye Terminator Cycle Sequencing Kit (Thermo Fisher Scientific) with an Applied Biosystems automated sequencer.

### *Candidatus* Riesia pediculicola housekeeping gene analysis

#### Primer design

In order to investigate the genotypic profile of the endosymbiotic bacteria, four standard PCR systems were designed targeting three *Candidatus* Riesia pediculicola housekeeping genes: *ftsZ, groEL* and two regions of *rpoB*. Four genomes of *Candidatus* Riesia pediculicola belonging to clade A and B lice deposited in the GenBank database (accession numbers CP012841, CP012843, CP012845, CP001085) [17, 18] were aligned using Muscle in MEGA7 software [24] and screened for conserved and discriminative genes.

Based on the variability in the available *Riesia* genomes, three housekeeping genes were selected as candidates for primer design. In order to find a suitable and conserved region for primers, two sequence fragments (approx. 100 bp) separated by a minimum of 500 bp for each gene were submitted to Primer3 software v. 0.4.0 (http://primer3.ut.ee/). The melting temperature of each primer was tested using the free online software programme Oligo Analyser 3.1 (https://eu.idtdna.com/calc/analyzer) [25]. Designed primers (Table 1) were then tested in silico, using the NCBI BLAST nucleotide sequence similarity tool (https://blast.ncbi.nlm.nih.gov/Blast.cgi).

**Table 1:** Details of designed primers for *Candidatus* Riesia pediculicola gene amplification

Endosymbiont of* Pediculus humanus*	Target gene	Primer sequences (5′–3′)	Tm	Fragment length (bp)
*Candidatus* Riesia pediculicola	*ftsZ*	*ftsZ*-196F_GGGAATTTCTGATCTTCTTCTGCG	56 °C	454
		*ftsZ*-650R_CTTTACTGGATGCTTTTGGYGC		
	*groEL*	*groEL*-560F_GATAGAGGTTATCTGTCTCC	50 °C	631
		*groEL*-1191R_GCAGCTCKAGTAGCATGTA		
	*rpoB*	*rpoB*-865F_ACCTGGTGATAAATCGTCTCC	54 °C	677
		*rpoB*-1542R_GAAAGAATCGTTCAGAAAGATCGG		
		*rpoB*-2619F_CAGCCATCTCTCCGATTGAACG	56 °C	466
		*rpoB*-3085R_CGATGGAAAGCTAATTTGTTCAGC		

Tm, Melting temperature

#### Housekeeping gene amplification

Prior to endosymbiotic DNA amplification, the designed primers were tested against a panel of negative controls consisting of the DNA of various bacterial species and arthropods (Additional file 2: Table S2). Once validated, 73 samples were randomly selected from the 126 lice to amplify *ftsZ, groEL* and the two *rpoB* regions of the endosymbiont (Table 1). Standard PCRs were performed as described for *cytb*, and amplicons were visualized on a 1.5% agarose gel.

### Data analysis

#### Phylogenetic analysis

In total, 28 lice specimens harbored common sequences for the *cytb* and the endosymbionte genes, but only 21 specimens had good quality sequences and were chosen for the phylogenetic analysis. The obtained *cytb* sequences were combined and compared with the worldwide *cytb* data previously reported and deposited in the GenBank database [8, 26]. Alignments were performed using MEGA7.0.26 software, and a maximum-likelihood (ML) tree was constructed using the Kimura2-parameter model under 1000 bootstrap replicates [27]. *Candidatus* Riesia pediculicola sequences were combined with data reported previously by Boyd et al. [16, 17] and were analysed as described for the *cytb* gene. Specifically, we used an Orthologous Average Nucleotide Identity Tool (OAT) [28] to define the overall similarities between the published *Candidatus* Riesia pediculicola genomes reported previously by Boyd et al. [17]. We also performed a Procrustes Approach to Cophylogenetic (PACo) analysis [29] using R [30] with 10,000 permutations, to test the dependence of the endosymbionte phylogeny on that of the louse clades by superimposing principal coordinates generated from the phylogenetic distance matrixes of *Candidatus* Riesia pediculicola and *P. humanus*.

## Results

### Lice clade identification

In this study, we collected 126 head lice and body lice worldwide (Additional file 1: Table S1) and selected 110 (87%) and 16 (13%), respectively, from these two populations for clade determination. qPCR assay of 125 of the 126 specimens showed that 76 (61%) of the lice belonged to the most prevalent clade, clade A, with various origins, including West Africa (17/76, 22.4%), Central Africa (10/76, 13.2%), North Africa (15/76, (9.7%), South Asia (24/76, 31.6%) and Europe (2/76, 2.6%), as well as reared lice used in the study as reference (8/76, 10.5%). Of the remaining lice specimens tested, 8/125 (6.3%) were genotyped as clade D; all these specimens were collected in the African continent, with six (75%) and two (25%) collected from Central and West Africa, respectively. Further, 39/125 (31.2%) of the analysed specimens belonged to clades C/E, with worldwide distribution: 21/39 (53.8%) and 11/39 (28.2%) specimens with Clades C/E were collected from West and Central Africa, respectively, and 7/39 (18%) were collected from Europe. Finally, only 2/125 (1.5%) samples belonged to clade B; these were collected in South Asia (Pakistan).

Standard PCR and sequencing of the *cytb* gene of 46/126 (36.5%) samples revealed that 12/46 (26.1%) belonged to clade A, 6/46 (13%) belonged to clade D, 2/46 (4.3%) belonged to clade B and only 1/46 (2.2%) belonged to the novel clade, clade F. In addition, 4/46 (8.7%) specimens belonged to clade C, while 21/46 (45.7%) of lice belonged to clade E (Table 2). Phylogenetical analysis was performed on 21 representative samples of all lice clades and origins.

**Table 2:** Results of mitochondrial analysis and *Candidatus* Riesia pediculicola housekeeping genes of human lice samples

Origin	Country	Number of lice	Clade mitochondrial analysis		*Candidatus* Riesia pediculicola housekeeping gene analysis (number of lice–qPCR/clade type)			
			Number of lice-qPCR/clade type	Number of lice-standard PCR/clade type	*ftsZ* gene	*groEL* gene	*rpoB*-1 gene	*rpoB*-2 gene
West Africa	Senegal	10	3/A; 2/D; 5/C	2/A; 2/D; 4/E	2/A; 2/D; 4/E	2/A; 2/D; 3/E	2/D; 1/E	2/A 2/D
	Guinea	6	6/C	6/E	6/E	6/E	6/E	6/E
East Africa	Ethiopia	3	3/C	2/C	3/C	3/C	2/C	2/C
Central Africa	Gabon	1	1/C	1/C	1/C	1/C	1/C	1/C
	Democratic Republic of the Congo	6	4/D; 2/C	4/D; 2/E	4/D; 2/E	3/D; 2/E	3/D; 1/E	3/D; 2/E
	Congo Brazzaville	3	3/C	NT	NT	NT	3/C	NT
North Africa	Algeria	3^a^	3/A	3/A	2/A	2/A	1/A	2/A
	Morocco	6	6/A	2/A	6/A	6/A	6/A	6/A
South Asia	India	13	13/A	2/A	11/A	10/A	12/A	11/A
	Pakistan	12	11/A; 2/B	2/A; 2/B	10/A; 2/B	11/A; 2/B	11/A; 2/B	11/A; 2/B
Europe	France	3	2/C	1/C; 1/F	2/C; 1/F	2/C; 1/F	1/C; 1/F	1/F
USA	Orlando strain	7^a^	7/A	2/A	6/A	6/A	6/A	6/A

NT, Not tested

^a^Body lice

### Phylogenetic relationship between the endosymbiont and their lice host

To determine the phylogenetic profile of *Candidatus* Riesia pediculicola, we selected 73/126 (57.9%) lice specimens for DNA amplification and sequencing of three housekeeping genes (*ftsZ, groEL* and *rpoB*). For *ftsZ*, *groEL* and the first and second regions of the *rpoB*, we were able to generate all four fragments in 46/73 (63%) selected louse sequences: 65/73 (89%), 62/73 (84.9%), 60/73 (82.2%) and 57/73 (78.1%) sequences, respectively (Table 1). However, 27/46 (58.7%) of the endosymbionts were in clade A lice collected worldwide [7/27 (26%) North Africa; 16/27 (59.3%) South Asia; 4/27 (14.7%) Orlando strain]. Of these 46 sequences, four (8.6%) belonged to clade D lice and originated from West and Central Africa; two (4.4%) specimens were clade B endosymbionts collected in South Asia (Pakistan); one (2.2%) belonged to the novel clade F collected in Europe (France); and four (8.7%) were clade C endosymbionts collected in East Africa. Finally, for its sister clade E, 8/46 (17.4%) specimens were collected from West and Central Africa (Table 2).

**Fig. 1:**
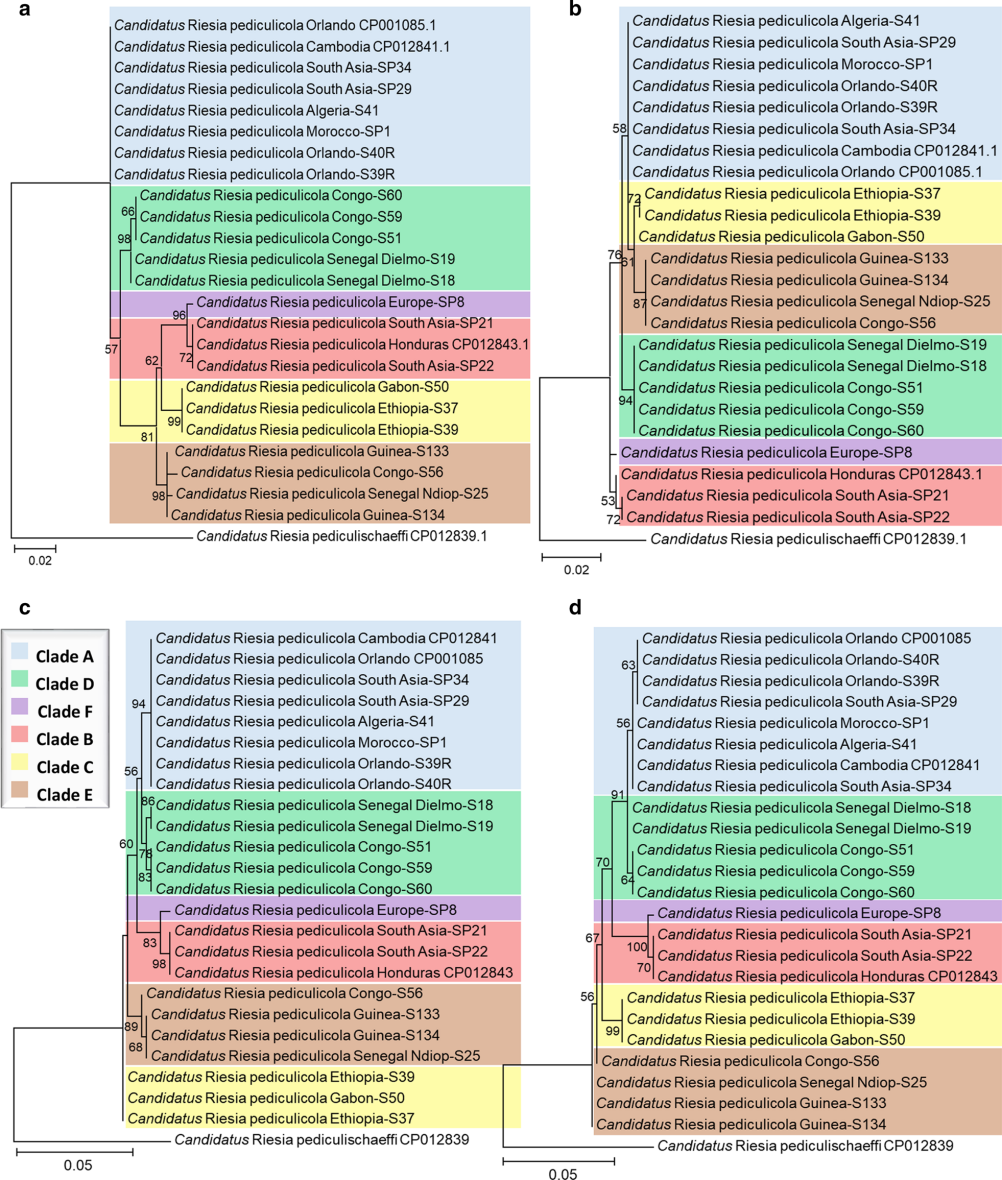
ML phylogenetic trees of the *Pediculus humanus* endosymbiont housekeeping genes, distributed in six divergent clades. **a** ML tree of a 454-bp fragment of the *ftsZ* gene, **b** ML tree of a 631-bp fragment of the *groEL* gene, **c**, **d** ML tree of the 677- and 466-bp fragments of the *rpoB* gene (*rpoB*-1 and *rpoB*-2, respectively). Phylogenetic inference was conducted in MEGA 7 using the ML method under the Kimura 2-parameter with 1000 bootstrap replicates.* Abbreviations*: ML, Maximum likelihood

**Fig. 2:**
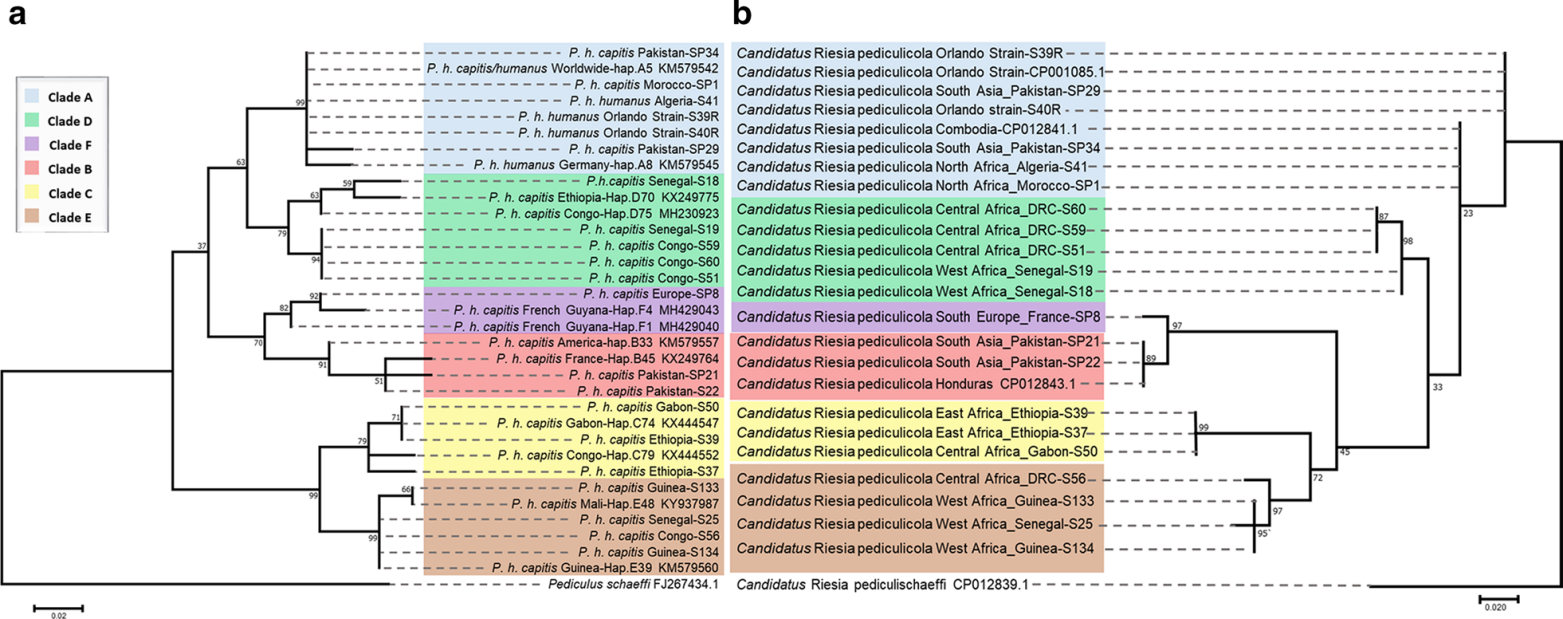
Phylogenetic tree of the *cytb* mitochondrial gene of *P. humanus* (**a**) projected with the concatenated *ftsZ* and *rpoB* (*rpoB*-2) genes (848 bp) of *Candidatus* Riesia pediculicola (**b**), showing the relationship and co-evolution of the endosymbiont dependently on the mitochondrial clades of their host. Phylogenetic inference was conducted in MEGA 7 using the ML method under the Kimura 2-parameter with 1000 bootstrap replicates

**Fig. 3:**
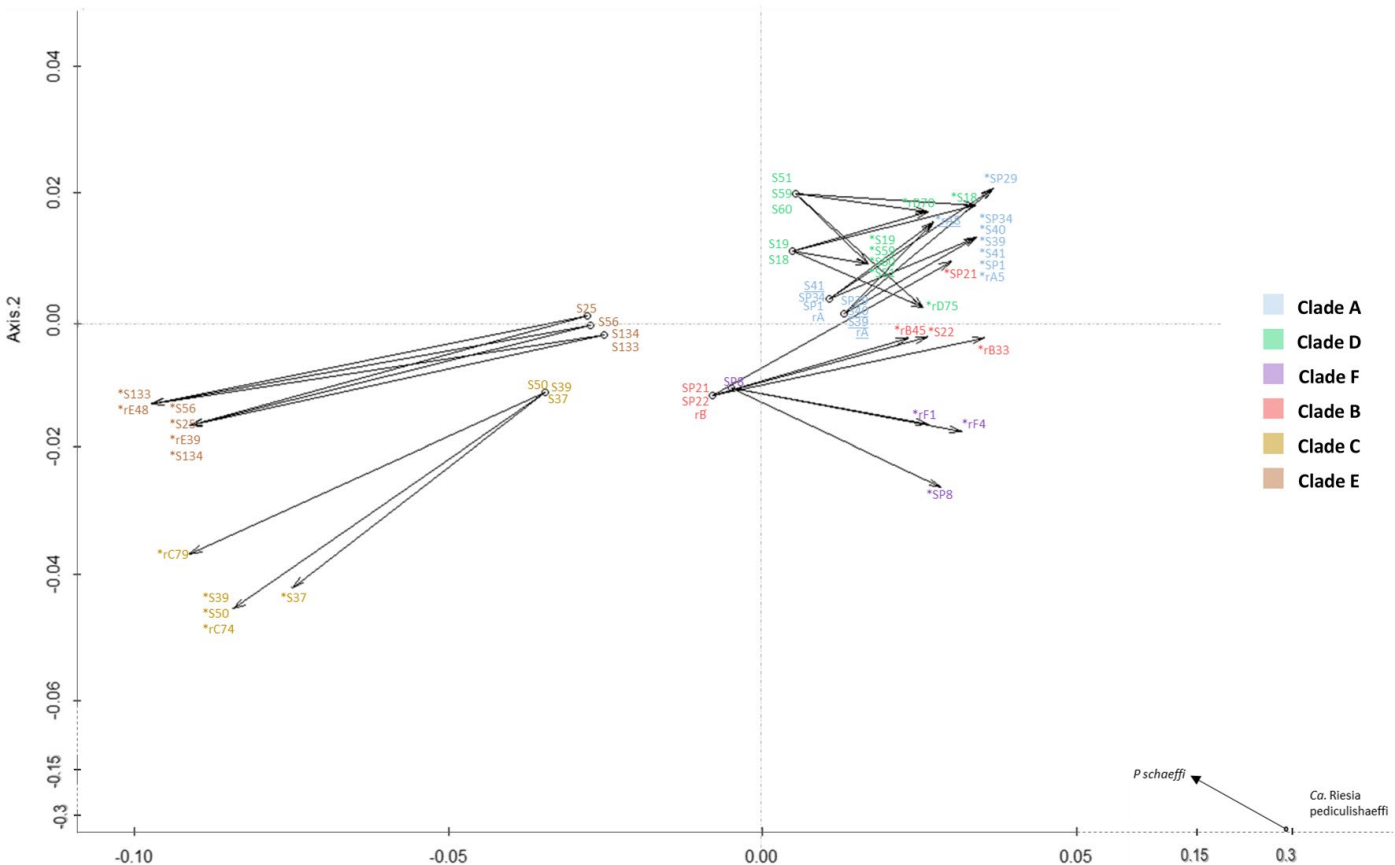
Procrustean superimposition plot of *Candidatus* Riesia pediculicola and *P. humanus*. The extended principal coordinate matrices (*X* and* Y*) are centred by mean column vectors and subjected to Procrustes analysis. The configuration of the endosymbiont (dots) has been rotated and scaled to fit the lice configuration (arrowheads). Asterisk indicates lice samples, underlining indicates body lice.* Abbreviations*: r, Reference sequences from GenBank; see phylogenetic trees in Figs. 1 and 2 for sample abbreviations

Maximum likelihood phylogenetic trees were constructed for the housekeeping genes, including 21 samples already analysed for the *cytb* gene. Interestingly, louse endosymbionts from each mt host clade clustered in a separate group (Fig. 1a, d), while the phylogeny based on the endosymbiotic *ftsZ* and the second region of *rpoB* (*rpoB*-2) genes followed that of *cytb*, which is not the case for the *groEL* and first region of *rpoB* (*rpoB*-1) genes (Fig. 1b, c). To further investigate the present congruent phylogenies, we proceeded to concatenate *ftsZ* and *rpoB-2* sequences (848 bp of the final fragment), and a phylogenetic tree was generated and compared to that of *cytb*. We noted that the concatenated 848-bp fragment of different *Candidatus* Riesia pediculicola harboured a higher bootstrap value and grouped in clades which were almost perfect when compared to the one gene-based tree. In addition, the clustering of sister clades was also visible for the B–F and C–E clades, mimicking the mt phylogeny of *P. humanus* (Fig. 2). Furthermore, PACo analysis showed a significant co-phylogenetic congruence between *Candidatus* Riesia pediculicola and *P. humanus* phylogenies across all clades. These results indicated a sum of squared residuals (*m*^2^) of 0.38 (*P* < 0.001) (Fig. 3).

The degree of genomic similarity of the deposited endosymbiont genomes of clade A and B lice [17] strongly support our results. A higher similarity (99.97%) was observed between *Candidatus* Riesia pediculicola of clade A head and body lice*,* with a lower similarity (97.85%) observed between clade A and B *P. h. capitis* specimens (Additional file 3: Figure S1).

## Discussion

In this study, we highlighted the codivergence of the endosymbiont *Candidatus* Riesia pediculicola and the mt clades of their *P. humanus* host. Louse endosymbionts were first reported in the 1920s, and they were subsequently successfully characterized in various histological, embryonic, experimental and nutritional studies [10]. This obligate intracellular primary endosymbiont is fully and uniquely attached to its host [16]. Its major role resides in providing essential B vitamins that are crucial for the survival of the louse and is lacking in the delivered blood meals [12, 31]. This bacterium has never been isolated in pure axenic culture, but studies employing molecular techniques suggest a polyphyletic origin [32]. Endosymbiotic microorganisms are generally associated with diverse arthropods, such as *Buchnera* of aphids, *Carsonella* of psyllids, *Portiera* of whiteflies, *Sulcia* of many homopterans, *Baumannia* of sharpshooters, *Blochmannia* of carpenter ants and *Nardonella* of weevils, as well as with bloodsucking insects, such as *Wigglesworthia* of tsetse flies [33, 34]. Their endosymbiont phylogeny generally mirrors the host phylogeny, indicating a stable and intimate host–symbiont association over time. This is also the case of the *Nycteribiidae* family of bat flies, which is involved in ectoparasitic blood-feeding on bats: phylogeny of the endosymbiont *Candidatus Aschnera chinzeii* clades shows co-speciation over the evolutionary course of the *Nycteribiidae* family [35]. Allen et al. dated the divergence between the *Riesia* and *Arsenophonus* (endosymbiont of *Lipoptena cervi*) clades at 13–25 million years ago [36]. Furthermore,* 16S* rDNA sequences confirmed a strict coevolution between the endosymbionts of *Anoplura* (i.e. *Haematopinus* sp. of ungulates*, Solenoptes* sp. of cattle, *Pediculus* sp. of hominids and *Polyplax* sp. of rodents) and *Rhyncophthirinan* (*Haematomyzus* sp. of Asian elephants) genera, with the endosymbiont sequences forming five separate monophyletic branches, each composed from only on louse genus [37]. Finally, the gene content of the *Columbicola wolffhuegeli* (endosymbiont of Pied Imperial Pigeon louse) was so similar to the gene content of the *Candidatus* Riesia pediculicola, based on the phylogenetic tree, that the human head louse and *C. wolffhuegeli* acquired their endosymbionts independently [38]. These findings suggest that every louse group has its own endosymbiont.

We demonstrated the evolutionary phylogenetic relationship that links *Candidatus* Riesia pediculicola to their host mt clades. Individual phylogenetic trees based on *ftsZ*, *rpoB-2* and their concatenated genes enabled an identical endosymbiotic clusterization depending on human lice clades. The endosymbiont phylogeny perfectly mirrored the host insect phylogeny, suggesting strict vertical transmission and host–symbiont co-speciation during the evolutionary course of the human louse. These data will allow the classification of human louse clades through their endosymbiotic bacteria based on the *ftsZ* and *rpoB*-2 genes. The slight discordance of *rpoB*-1- and, in particular, *groEL*-based trees (Fig. 1b, c) with host *cytB*-based topologies may be due to the lack of sufficient informative characters. The concatenated tree of the four gene fragment analysed, however, is in perfect agreement with thehost tree (Fig. 2). We further investigated this phenomenon by constructing a ML phylogenetic tree of human, gorilla and chimpanzee lice and their endosymbionts; however, the paucity of available sequences did not allow us to conclude if there is a probable gene tree conflict (Additional file 4: Figure S2). While we observed a clear characterization of the lice clades for the first region of the *rpoB*-1 gene, differences regarding clades E and C were observed. The slightly incongruent trees obtained within the same gene can be explained by a difference in the mutation level between these two regions. Our findings need further investigation by sequencing and analysing the endosymbionts’ whole genomes within all human lice clades to better establish the evolutionary time courses within their hosts.

## Conclusion

Based on phylogenetic and genomic analyses, we have highlighted the co-evolutionary relationship between *Candidatus* Riesia pediculicola and their host mt clades. Our results unequivocally indicate that louse endosymbionts have experienced a similar co-evolutionary history and that human lice clades can be determined by their endosymbiotic bacteria based on their *ftsZ* and *rpoB* housekeeping genes. In future studies, further robust phylogenetic examination of all endosymbiont genome clades will be fundamental to a better understanding of the evolution of *Candidatus* Riesia pediculicola depending on the mt divergence of their hosts. However, it is crucial to isolate and identify this bacterium in order to evaluate the effectiveness of a drug treatment targeting the louse endosymbiont.

## Supplementary Information

**Additional file 1: Table S1.** Results of mitochondrial analysis of human lice samples and *Candidatus* Riesia pediculicola housekeeping gene analysis.

**Additional file 2: Table S2.** Negative control DNA used for sensitivity and specificity determination of designed oligonucleotides.

**Additional file 3: Figure S1.** Heatmap generated according to OrthoANI values calculated using Orthologous Average Nucleotide Identity Tool (OAT) software (https://www.ezbiocloud.net/tools/orthoani) to measure the overall similarity between the genomes of *Candidatus* Riesia sp. strains and other related members of the *Riesia* genus.* Abbreviations*: BL, body lice; HapA, *Candidatus* Riesia pediculicola from *P. humanus* clade A; HapB, *Candidatus* Riesia pediculicola from *P. humanus* clade B; HL, head lice

**Additional file 4: Figure S2.** ML phylogenetic tree of primates lice (left) and their endosymbions “*Ca*. Riesia sp.” (right). Phylogenetic inference was conducted in MEGA 7 using the maximum likelihood method under the Kimura 2-parameter with 1000 bootstrap replicates.

## Data Availability

The sequences of the *Candidatus* Riesia pediculicola housekeeping genes referred to in this article and the attributed GenBank accession numbers are as follows. *ftsZ* gene: clade A (Orlando-MW588459, Senegal-MW588463), clade D (Congo-MW588466, Senegal-MW588464), clade B (Pakistan-MW588462), clade F (France-MW588460), clade C (France-MW58846) and clade E (Congo-MW588465, Guinea-MW588458). The second region of the endosymbiont *rpoB* gene: clade A (Orlando-MW588485, India-MW588486, Algeria-MW588487), clade D (Senegal-MW588488, Congo-MW588489); clade B (Pakistan-MW588491), clade F (France-MW588490), clade C (Ethiopia-MW588492) and clade E (Congo-MW588494, Guinea-MW588493).
