# Supplementary material for: Phylogenetic relationship between the endosymbiont “Candidatus Riesia pediculicola” and its human louse host
Source: Parasit Vectors. 2022 Mar 5;15:73. doi: 10.1186/s13071-022-05203-z (PMC8898481; doi:10.1186/s13071-022-05203-z)
Supplement: Supplementary file 1 — Additional file 1: Table S1. Results of mitochondrial analysis of human lice samples and Candidatus Riesia pediculicola housekeeping gene analysis. [file 13071_2022_5203_MOESM1_ESM.docx]

**Table S1.** Results of mitochondrial analysis of human lice samples as well as *Candidatus* Riesia pediculicola housekeeping gene analysis

| **Origin** | **Country/ Year** | **Samples ID** | **Ecotype** | **Clade mitochondrial analysis** | | ***Candidatus* Riesia pediculicola housekeeping gene analysis** | | | |
| --- | --- | --- | --- | --- | --- | --- | --- | --- | --- |
|  |  |  |  | **qPCR clade** | **St PCR *Cytb*** | ***ftsZ* gene** | ***groEL* gene** | ***rpoB*-1 gene** | ***rpoB*-2 gene** |
| **West Africa** | **Dakar-Senegal** | 1 | HL | A | NT | NT | NT | NT | NT |
|  |  | 2 | HL | A | NT | NT | NT | NT | NT |
|  |  | 3 | HL | A | NT | NT | NT | NT | NT |
|  | **Malika-Senegal** | 4 | HL | A | NT | NT | NT | NT | NT |
|  |  | 5 | HL | A | NT | NT | NT | NT | NT |
|  |  | 6 | HL | A | A | NT | NT | NT | NT |
|  |  | 7 | HL | C | E | NT | NT | NT | NT |
|  |  | 8 | HL | A | A | ✓ | ✓ | - | ✓ |
|  |  | 9 | HL | A | NT | NT | NT | NT | NT |
|  | **Dielmo 2010-Senegal** | 10 | HL | A | NT | NT | NT | NT | NT |
|  |  | 11 | HL | C | NT | NT | NT | NT | NT |
|  |  | 12 | HL | C | E | ✓ | - | ✓ | - |
|  |  | 13 | HL | C | NT | ✓ | ✓ | ✓ | - |
|  | **Dielmo 2014-Senegal** | 14 | HL | D | D | ✓ | ✓ | ✓ | ✓ |
|  |  | 15 | HL | D | D | ✓ | ✓ | ✓ | ✓ |
|  |  | 16 | HL | A | NT | NT | NT | NT | NT |
|  |  | 17 | HL | A | NT | NT | NT | NT | NT |
|  |  | 18 | HL | A | NT | NT | NT | NT | NT |
|  |  | 19 | HL | A | NT | NT | NT | NT | NT |
|  | **Ndiop 2010-Senegal** | 20 | HL | A | NT | ✓ | ✓ | - | ✓ |
|  |  | 21 | HL | A | NT | NT | NT | NT | NT |
|  | **Ndiop 2014-Senegal** | 22 | HL | A | A | ✓ | ✓ | - | ✓ |
|  |  | 23 | HL | C | E | ✓ | ✓ | ✓ | ✓ |
|  |  | 24 | HL | C | E | ✓ | ✓ | - | - |
|  |  | 25 | HL | C | E | ✓ | ✓ | - | - |
|  |  | 26 | HL | A | NT | NT | NT | NT | NT |
|  | **Guinea 2018** | 27 | HL | C | E | NT | NT | NT | NT |
|  |  | 28 | HL | C | E | NT | NT | NT | NT |
|  |  | 29 | HL | C | E | NT | NT | NT | NT |
|  |  | 30 | HL | C | E | NT | NT | NT | NT |
|  |  | 31 | HL | C | E | NT | NT | NT | NT |
|  |  | 32 | HL | C | E | NT | NT | NT | NT |
|  |  | 33 | HL | C | E | NT | NT | NT | NT |
|  |  | 34 | HL | C | E | ✓ | ✓ | ✓ | ✓ |
|  |  | 35 | HL | C | E | NT | NT | NT | NT |
|  |  | 36 | HL | C | E | ✓ | ✓ | ✓ | ✓ |
|  |  | 37 | HL | C | E | ✓ | ✓ | ✓ | ✓ |
|  |  | 38 | HL | C | E | ✓ | ✓ | ✓ | ✓ |
|  |  | 39 | HL | C | E | ✓ | ✓ | ✓ | ✓ |
|  |  | 40 | HL | C | E | ✓ | ✓ | ✓ | ✓ |
| **East Africa** | **Ethiopia** | 41 | HL | C | NT | ✓ | ✓ | - | - |
|  |  | 42 | HL | C | C | ✓ | ✓ | ✓ | ✓ |
|  |  | 43 | HL | C | C | ✓ | ✓ | ✓ | ✓ |
|  |  | 44 | HL | C | NT | NT | NT | NT | NT |
| **Central Africa** | **Gabon** | 45 | HL | C | C | ✓ | ✓ | ✓ | ✓ |
|  | **Democratic Republic of Congo** | 46 | HL | D | D | ✓ | ✓ |  | ✓ |
|  |  | 47 | HL | D | NT | NT | NT | NT | NT |
|  |  | 48 | HL | D | D | ✓ | - | - | - |
|  |  | 49 | HL | A | NT | NT | NT | NT | NT |
|  |  | 50 | HL | C | E | ✓ | ✓ | - | ✓ |
|  |  | 51 | HL | C | E | ✓ | ✓ | ✓ | ✓ |
|  |  | 52 | HL | A | NT | NT | NT | NT | NT |
|  |  | 53 | HL | A | NT | NT | NT | NT | NT |
|  |  | 54 | HL | D | D | ✓ | ✓ | ✓ | ✓ |
|  |  | 55 | HL | D | D | ✓ | ✓ | ✓ | ✓ |
|  |  | 56 | HL | D | NT | NT | NT | NT | NT |
|  | **Brazzaville** | 57 | HL | C | NT | - | - | ✓ | - |
|  |  | 58 | HL | C | NT | NT | NT | NT | NT |
|  |  | 59 | HL | A | NT | NT | NT | NT | NT |
|  |  | 60 | HL | A | NT | NT | NT | NT | NT |
|  |  | 61 | HL | A | NT | NT | NT | NT | NT |
|  |  | 62 | HL | A | NT | NT | NT | NT | NT |
|  |  | 63 | HL | A | NT | NT | NT | NT | NT |
|  |  | 64 | HL | C | NT | - | - | ✓ | - |
|  |  | 65 | HL | C | NT | - | - | ✓ | - |
|  |  | 66 | HL | A | NT | NT | NT | NT | NT |
|  |  | 67 | HL | A | NT | NT | NT | NT | NT |
| **North Africa** | **Algeria-2017** | 68 | BL | A | A | - | - | - | ✓ |
|  |  | 69 | BL | A | A | ✓ | ✓ | ✓ | ✓ |
|  |  | 70 | BL | A | NT | NT | NT | NT | NT |
|  |  | 71 | BL | A | A | ✓ | ✓ | - | - |
|  |  | 72 | BL | A | NT | NT | NT | NT | NT |
|  |  | 73 | BL | A | NT | NT | NT | NT | NT |
|  |  | 74 | BL | A | NT | NT | NT | NT | NT |
|  |  | 75 | BL | A | NT | NT | NT | NT | NT |
|  | **Morocco** | 76 | HL | A | A | ✓ | ✓ | ✓ | ✓ |
|  |  | 77 | HL | A | NT | NT | NT | NT | NT |
|  |  | 78 | HL | A | NT | ✓ | ✓ | ✓ | ✓ |
|  |  | 79 | HL | A | NT | ✓ | ✓ | ✓ | ✓ |
|  |  | 80 | HL | A | NT | ✓ | ✓ | ✓ | ✓ |
|  |  | 81 | HL | A | A | ✓ | ✓ | ✓ | ✓ |
|  |  | 82 | HL | A | NT | ✓ | ✓ | ✓ | ✓ |
| **South Asia** | **India** | 83 | HL | A | NT | ✓ | ✓ | ✓ | ✓ |
|  |  | 84 | HL | A | NT | ✓ | ✓ | ✓ | ✓ |
|  |  | 85 | HL | A | NT | ✓ | - | ✓ | - |
|  |  | 86 | HL | A | NT | - | - | ✓ | ✓ |
|  |  | 87 | HL | A | NT | - | ✓ | ✓ | ✓ |
|  |  | 88 | HL | A | NT | ✓ | ✓ | ✓ | ✓ |
|  |  | 89 | HL | A | A | ✓ | ✓ | ✓ | ✓ |
|  |  | 90 | HL | A | A | ✓ | ✓ | ✓ | ✓ |
|  |  | 91 | HL | A | NT | ✓ | ✓ | - | ✓ |
|  |  | 92 | HL | A | NT | ✓ | ✓ | ✓ | ✓ |
|  |  | 93 | HL | A | NT | ✓ | ✓ | ✓ | ✓ |
|  |  | 94 | HL | A | NT | ✓ | - | ✓ | - |
|  |  | 95 | HL | A | NT | ✓ | ✓ | ✓ | ✓ |
|  | **Pakistan** | 96 | HL | B | B | ✓ | ✓ | ✓ | ✓ |
|  |  | 97 | HL | B | B | ✓ | ✓ | ✓ | ✓ |
|  |  | 98 | HL | A | NT | ✓ | ✓ | ✓ |  |
|  |  | 99 | HL | A | NT | - | - | - | ✓ |
|  |  | 100 | HL | A | NT | ✓ | ✓ | ✓ | ✓ |
|  |  | 101 | HL | A | NT | ✓ | ✓ | ✓ | ✓ |
|  |  | 102 | HL | A | NT | ✓ | ✓ | ✓ | ✓ |
|  |  | 103 | HL | A | A | ✓ | ✓ | ✓ | ✓ |
|  |  | 104 | HL | A | NT | NT | NT | NT | NT |
|  |  | 105 | HL | A | NT | ✓ | ✓ | ✓ | ✓ |
|  |  | 106 | HL | A | NT | ✓ | ✓ | ✓ | ✓ |
|  |  | 107 | HL | A | NT | ✓ | ✓ | ✓ | ✓ |
|  |  | 108 | HL | A | A | ✓ | ✓ | ✓ | ✓ |
| **Europe** | **France** | 109 | HL | - | F | ✓ | ✓ | ✓ | ✓ |
|  |  | 110 | HL | C | NT | NT | NT | NT | NT |
|  |  | 111 | HL | C | NT | NT | NT | NT | NT |
|  |  | 112 | HL | A | NT | NT | NT | NT | NT |
|  |  | 113 | HL | C | NT | ✓ | ✓ | ✓ | - |
|  |  | 114 | HL | A | NT | NT | NT | NT | NT |
|  |  | 115 | HL | C | NT | NT | NT | NT | NT |
|  |  | 116 | HL | C | NT | NT | NT | NT | NT |
|  |  | 117 | HL | C | C | ✓ | ✓ | - | - |
|  |  | 118 | HL | C | NT | NT | NT | NT | NT |
| **USA** | **Orlando strain** | 119 | BL | A | NT | NT | NT | NT | NT |
|  |  | 120 | BL | A | NT | ✓ | ✓ | ✓ | ✓ |
|  |  | 121 | BL | A | NT | ✓ | ✓ | ✓ | ✓ |
|  |  | 122 | BL | A | NT | ✓ | ✓ | - | - |
|  |  | 123 | BL | A | A | ✓ | ✓ | ✓ | ✓ |
|  |  | 124 | BL | A | A | ✓ | ✓ | ✓ | ✓ |
|  |  | 125 | BL | A | NT | ✓ | ✓ | ✓ | ✓ |
|  |  | 126 | BL | A | NT | - | - | ✓ | ✓ |

**HL:** Head lice; **BL:** Body lice; **✓:** sequences obtained for housekeeping genes; **-:** positive PCR but no sequence obtained
