# Supplementary material for: Phylogenetic relationship between the endosymbiont “Candidatus Riesia pediculicola” and its human louse host
Source: Parasit Vectors. 2022 Mar 5;15:73. doi: 10.1186/s13071-022-05203-z (PMC8898481; doi:10.1186/s13071-022-05203-z)
Supplement: Supplementary file 2 — Additional file 2: Table S2. Negative control DNA used for sensitivity and specificity determination of designed oligonucleotides. [file 13071_2022_5203_MOESM2_ESM.docx]

**Table S2.** Negative control DNA used for sensitivity and specificity determination of designed oligonucleotides

| **N°** | **Organism** | **Species** |
| --- | --- | --- |
| 1 | Bacteria | *Acinetobacter baumannii* |
| 2 | Bacteria | *Acinetobacter sp.* |
| 3 | Tick | *Amblyomma variegatum* |
| 4 | Bacteria | *Anaplasma phagocytophilum* |
| 5 | Bacteria | *Asaia bogorensis* |
| 6 | Piroplasm | *Babesia canis* |
| 7 | Bacteria | *Bartonella henselae* |
| 8 | Bacteria | *Bartonella quintana* |
| 9 | Bacteria | *Borrelia recurrentis* |
| 10 | Bacteria | *Borrelia theileri* |
| 11 | Bedbugs | *Cimex lectularius* |
| 12 | Bacteria | *Citrobacter koseri* |
| 13 | Bacteria | *Coxiella burnetii* |
| 14 | Flea | *Ctenocephalides felis* |
| 15 | Protozoa | *Dirofilaria immitis* |
| 16 | Bacteria | *Ehrlichia canis* |
| 17 | Bacteria | *Enterobacter aerogenes* |
| 18 | Bacteria | *Enterococcus faecium* |
| 19 | Bacteria | *Gardnerella vaginalis* |
| 20 | Bacteria | *Haemophilus influenzae* |
| 21 | Protozoa | *Hepatozoon canis* |
| 22 | Human | HL60 cell line *(Homo sapiens)* |
| 23 | Tick | *Hyalomma marginatum* |
| 24 | Protozoa | *Leishmania donovani* |
| 25 | Protozoa | *Leishmania major* |
| 26 | Protozoa | *Leptomonas saimouri* |
| 27 | Protozoa | *Plasmodium falciparum* |
| 28 | Protozoa | *Plasmodium malariae* |
| 29 | Protozoa | *Plasmodium ovale* |
| 30 | Protozoa | *Plasmodium vivax* |
| 31 | Tick | *Rhipicephalus microplus* |
| 32 | Bacteria | *Rickettsia conorii* |
| 33 | Bacteria | *Rickettsia felis* |
| 34 | Bacteria | *Rickettsia massiliae* |
| 35 | Bacteria | *Rickettsia montanensis* |
| 36 | Bacteria | *Rickettsia typhi* |
| 37 | Bacteria | *Salmonella enterica* |
| 38 | Bacteria | *Staphylococcus aureus* |
| 39 | Bacteria | *Staphylococcus haemolyticus* |
| 40 | Bacteria | *Staphylococcus hominis* |
| 41 | Bacteria | *Stenotrophomonas maltophilia* |
| 42 | Bacteria | *Streptococcus agalactiae* |
| 43 | Bacteria | *Streptococcus pneumoniae* |
| 44 | Bacteria | *Streptococcus pyogenes* |
| 45 | Piroplasm | *Theileria equi* |
| 46 | Bacteria | *Treponema pallidum* |
| 47 | Protozoa | *Trypanosoma evansi* |
| 48 | Protozoa | *Trypanosoma gambiense* |
| 49 | Protozoa | *Trypanosoma vivax* |
| 50 | Bacteria | *Wolbachia* sp. |
| 51 | Bacteria | *Yersinia pestis* |
