# Supplementary figures and images for: Phylogenetic relationship between the endosymbiont “Candidatus Riesia pediculicola” and its human louse host
Source: Parasit Vectors. 2022 Mar 5;15:73. doi: 10.1186/s13071-022-05203-z (PMC8898481; doi:10.1186/s13071-022-05203-z)

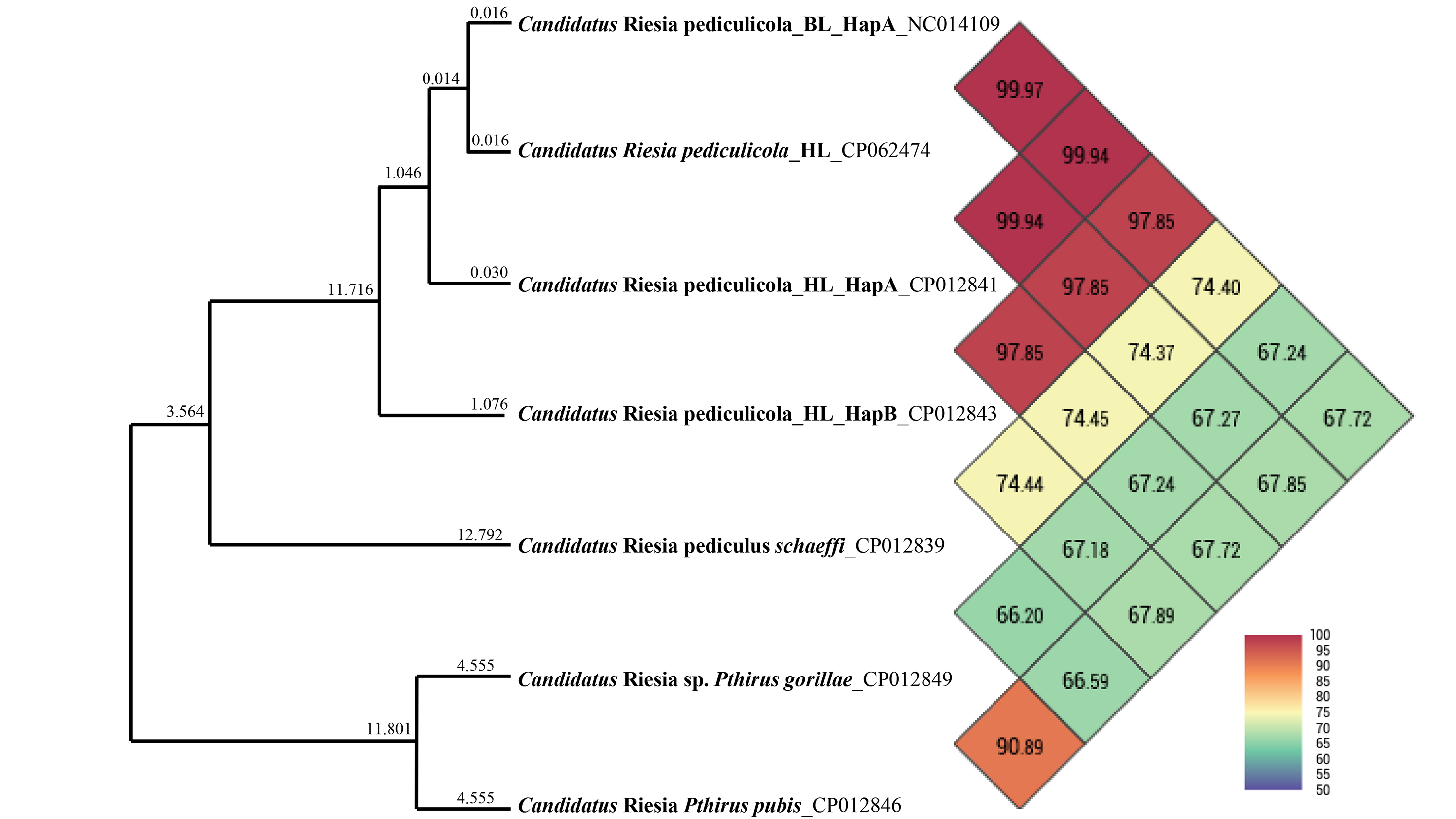

Supplement: Supplementary file 3 — Additional file 3: Figure S1. Heatmap generated according to OrthoANI values calculated using Orthologous Average Nucleotide Identity Tool (OAT) software (https://www.ezbiocloud.net/tools/orthoani) to measure the overall similarity between the genomes of Candidatus Riesia sp. strains and other related members of the Riesia genus. Abbreviations: BL, body lice; HapA, Candidatus Riesia pediculicola from P. humanus clade A; HapB, Candidatus Riesia pediculicola from P. humanus clade B; HL, head lice [file 13071_2022_5203_MOESM3_ESM.tif]

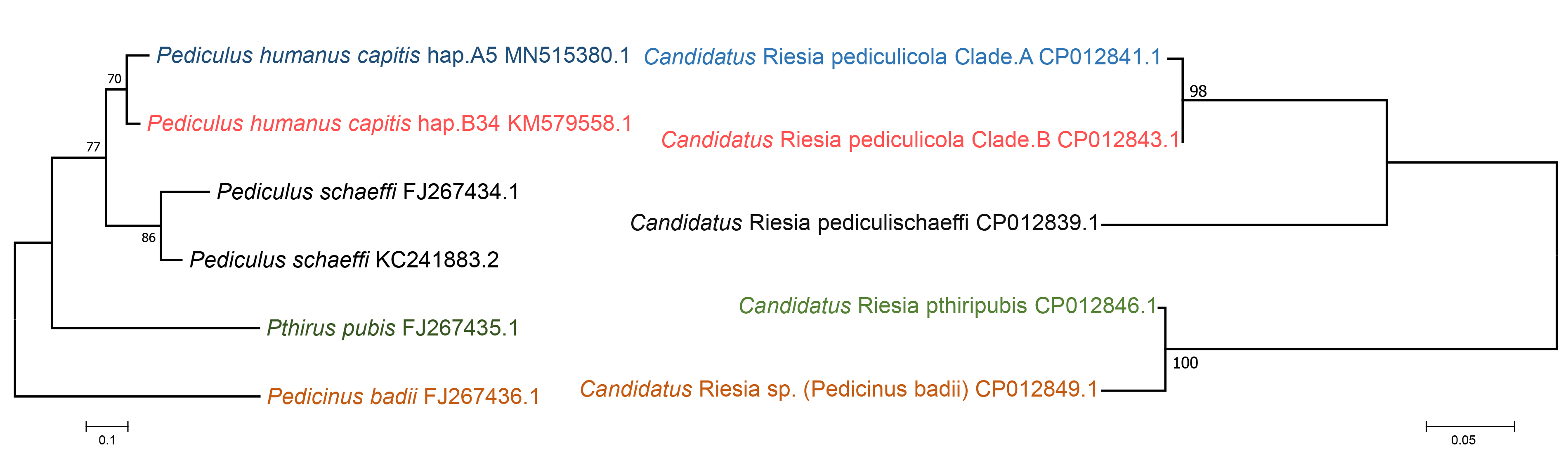

Supplement: Supplementary file 4 — Additional file 4: Figure S2. ML phylogenetic tree of primates lice (left) and their endosymbions “Ca. Riesia sp.” (right). Phylogenetic inference was conducted in MEGA 7 using the maximum likelihood method under the Kimura 2-parameter with 1000 bootstrap replicates. [file 13071_2022_5203_MOESM4_ESM.jpg]
